# Cancer-specific functional profiling in microsatellite-unstable (MSI) colon and endometrial cancers using combined differentially expressed genes and biclustering analysis

**DOI:** 10.1097/MD.0000000000033647

**Published:** 2023-05-12

**Authors:** Woong Na, Il Ju Lee, Insong Koh, Mihye Kwon, Young Soo Song, Sung Hak Lee

**Affiliations:** a Department of Pathology, H Plus Yangji Hospital, Seoul, South Korea; b Department of Pathology, College of Medicine, Hanyang University, Seoul, South Korea; c Department of Biomedical Informatics, Graduate School of Biomedical Science & Engineering, Hanyang University, Seoul, South Korea; d Department of Internal Medicine, College of Medicine, Konyang University, Daejeon, South Korea; e Department of Pathology, College of Medicine, Konyang University, Daejeon, South Korea; f Department of Pathology, College of Medicine, Catholic University, Seoul, South Korea.

**Keywords:** biclustering, colon cancer, differentially expressed gene, endometrial cancer, gene expression profiling, microsatellite instability

## Abstract

Microsatellite-unstable (MSI) cancers have distinct genetic and clinical features from microsatellite-stable cancers, but the molecular functional differences between MSI cancers originating from different tissues or organs have not been well studied because the application of usual differentially expressed gene (DEG) analysis is error-prone, producing too many noncancer-specific normally functioning genes. To maximize therapeutic efficacy, biomarkers reflecting cancer-specific differences between MSI cancers of different tissue origins should be identified. To identify functional differences between MSI colon and endometrial cancers, we combined DEG analysis and biclustering instead of DEG analysis alone and refined functionally relevant biclusters reflecting genuine functional differences between the 2 tumors. Specifically, using The Cancer Genome Atlas and genome-tissue expression as data sources, gene ontology (GO) enrichment tests were performed after routinely identifying DEGs between the 2 tumors with the exclusion of DEGs identified in their normal counterparts. Cancer-specific biclusters and associated enriched GO terms were obtained by biclustering with enrichment tests for the preferences for cancer type (either colon or endometrium) and GO enrichment tests for each cancer-specific bicluster, respectively. A novel childness score was developed to select functionally relevant biclusters among cancer-specific biclusters based on the extent to which the enriched GO terms of the biclusters tended to be child terms of the enriched GO terms in DEGs. The selected biclusters were tested using survival analysis to validate their clinical significance. We performed multiple sequential analyses to produce functionally relevant biclusters from the RNA sequencing data of MSI colon and endometrial cancer samples and their normal counterparts. We identified 3066 cancer-specific DEGs. Biclustering analysis revealed 153 biclusters and 41 cancer-specific biclusters were selected using Fisher exact test. A mean childness score over 0.6 was applied as the threshold and yielded 8 functionally relevant biclusters from cancer-specific biclusters. Functional differences appear to include gland cavitation and the TGF-β receptor, G protein, and cytokine pathways. In the survival analysis, 6 of the 8 functionally relevant biclusters were statistically significant. By attenuating noise and applying a synergistic contribution of DEG results, we refined candidate biomarkers to complement tissue-specific features of MSI tumors.

## 1. Introduction

Microsatellite instability (MSI) is a distinct genetic condition that leads to carcinogenesis in a subset of tumors, particularly colorectal carcinoma (CRC) and endometrial carcinoma.^[1–7]^ Identifying the occurrence of MSI is of particular clinical importance, given that immune checkpoint inhibitors can be administered to these patients irrespective of the histology or tissue of origin.^[2,8–11]^ In addition, mutational profiles differ between MSI and microsatellite-stable (MSS) tumors.^[12]^ MSI-CRC has been reported to have a higher tumor mutation burden and a lower copy number than MSS tumors.^[13]^ Analysis of the differential gene expression data for MSI-CRC has indicated that the most frequently altered functional classes are cell cycle, DNA replication, recombination, repair, gastrointestinal disease, and immune response.^[14]^ Likewise, compared with patients with MSS CRCs, those with MSI-high (H) CRC have been found to be characterized by a higher expression of immune-related genes.^[15]^

Although different MSI tumors are characterized by similar carcinogenesis irrespective of the tissue of origin, there appear to be marked differences in prognosis and the response to therapy, depending on the tissue of origin,^[3,6,7,9–11]^ thereby indicating the presence of underlying tissue-specific carcinogenic mechanisms with large effects. Accordingly, biomarkers reflecting tissue-specific carcinogenesis should ideally be identified to enhance current checkpoint inhibitor-based immunotherapy and to develop new therapeutic strategies that can be applied to maximize therapeutic responses.^[1,16]^

Identifying differentially expressed genes (DEGs) via gene expression profiling is a routine approach used in oncological studies.^[17,18]^ Consequently, we have adopted these methods to investigate tissue-specific carcinogenesis in MSI cancers (colon and endometrial). The methodologies typically applied for the identification of DEGs tend to be very effective when analyzing a single type of condition, such as tumor versus normal tissues. However, many researchers are aware of the methodological limitations of this approach when >2 types of interacting conditions are involved.^[19]^ In our studies, most of the genes identified using standard DEG identification methodologies might be normally functioning tissue-specific genes that are unrelated to carcinogenesis.^[19]^ These issues were addressed in the Cancer Genome Atlas (TCGA) PanCancer project, in which the applicability of methodologies based on orthogonal partial least squares-discriminant analysis was assessed as alternatives to the standard procedures.^[19]^ Biologically relevant DEGs were identified, and many normal tissue-specific genes were removed. If the standard DEG identification procedures were applied, such as DeSeq2 or edgeR, it was found that the list of genes finally obtained was very different and unstable.^[20,21]^ However, DEG identification in the context of >1 type of condition remains methodologically complex, and the optimal threshold is difficult to determine. Moreover, if a nonnegligible degree of molecular heterogeneity in a sample cohort is anticipated, which is a frequent occurrence in studies investigating tumor samples across different tissue origins, DEGs alone would be insufficient as the main outcome of gene expression profiling.

Biclustering, a technique that is widely used in functional profiling, is a data-mining technique that yields closely related subsets of genes and samples. Data modulated by biclustering can be used as essential building blocks for investigating molecular heterogeneity.^[22]^ Unlike clustering, which divides genes or samples into several mutually exclusive subsets, biclustering operates on both genes and samples and produces multiple overlapping biclusters. Given that each bicluster is a distinct fraction of the entire dataset and is potentially functionally correlated, identifying functionally relevant biclusters can partly replace the procedure of DEG identification. However, the typically adopted biclustering procedures produce only a small proportion of functionally relevant biclusters, along with considerable amounts of irrelevant products that require further extensive functional filtering.

Formalized knowledge bases such as gene ontology (GO) are particularly useful in evaluating the functional relevance of biclusters. GO is a highly organized formal representation of gene products developed and maintained by the GO Consortium.^[23]^ Most gene expression profiles are currently analyzed using GO. By investigating the enriched GO terms of a bicluster and combining these results with those obtained based on DEG analysis, a more refined set of biclusters, which are potential biomarkers of interest, can be obtained.

In this study, we examined the cancer-specific functions of 2 main types of MSI cancers (colon cancer and endometrial cancer) using gene expression data from TCGA. To overcome the typical limitations of error-prone DEG identification procedures, we developed a novel method that combines biclustering and DEG identification using enriched GO terms. These refined sets of biclusters reflected more cancer-specific functions in MSI colon or endometrial cancers, and we assessed their clinical significance as potential biomarkers based on survival analysis.

## 2. Methods

### 2.1. Data collection

**Figure 1.**
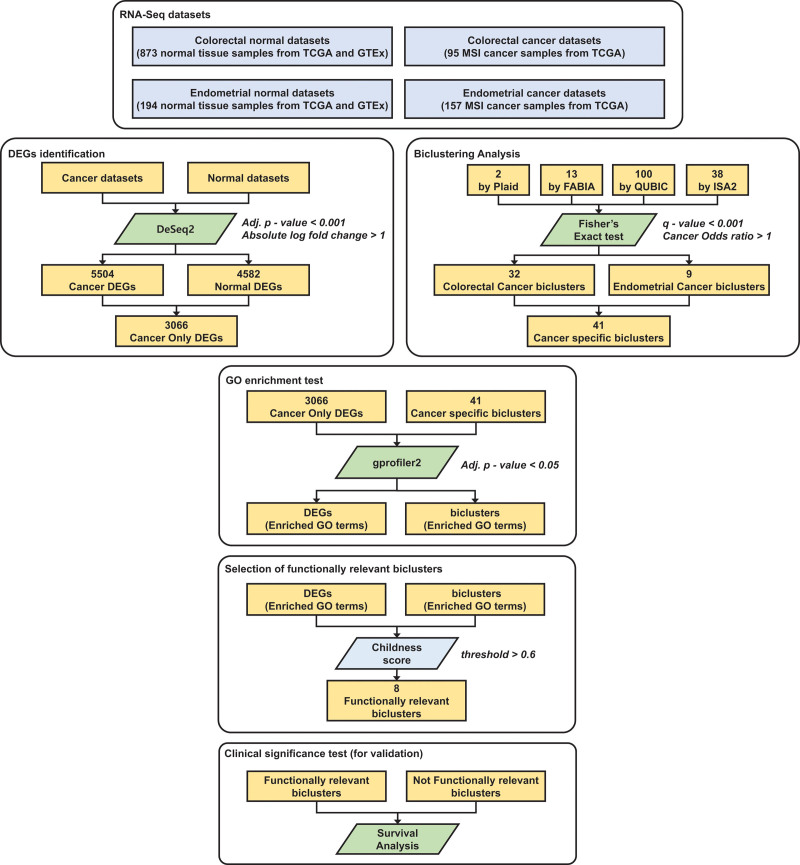
Overall workflow of the study. The direction of the workflow is top to down. Data are represented as rectangles and methods or tools as parallelograms. If a process contains >1 subcomponent, they are represented as rounded rectangles. adj. *P* value = adjusted *P* value using false discovery rate, DEG = differentially expressed gene, GO = gene ontology, GTEx = genotype-tissue expression, TCGA = the Cancer Genome Atlas.

The workflow of this study is illustrated in Figure 1. Cleansed and refined RNA sequencing data for TCGA CRC, endometrial carcinoma, and genotype-tissue expression (GTEx) of colon and endometrial samples were downloaded from the Recount3 project site using the Recount3 package with the corresponding clinicopathological data.^[24]^ In accordance with the policies of TCGA and GTEx, neither ethical approval nor patient consent was necessary for this study. The Recount3 project aims to provide uniformly processed analysis-ready RNA sequencing data from diverse resources, including TCGA and GTEx, using optimized pipelines.^[24]^ Having initially selected the MSI-H cancer and TCGA normal samples (colon and endometrium), these were subsequently combined with GTEx samples, yielding a total of 1319 samples: 873 normal colorectal tissue, 95 colorectal MSI cancers, 194 normal endometrial tissue, and 157 endometrial MSI cancers. Using principal component analysis (PCA), we confirmed that the TCGA normal samples could be combined with GTEx samples without further processing for our research goals, as the bias between GTEx and TCGA normal samples would have a minimal influence on the final results (See Supplementary Figure S1, Supplemental Digital Content, http://links.lww.com/MD/I902, which illustrates the PCA of gene expression in TCGA normal and GTEx samples).

### 2.2. DEGs

The identification of genes characterized by differential expression between the colon and endometrial cancers of the MSI subtype was performed using DESeq2-based methods.^[20]^ Initially, each DEG between colon and endometrial MSI cancers and between normal colon and endometrial tissues was identified based on the threshold criteria of an adjusted *P* value < 0.001 and an absolute log fold change > 1. DEGs in normal tissues were excluded from those in tumors to obtain cancer-specific DEGs. Although these DEG identification procedures may have limitations, the crude DEG lists were compensated for in the following steps: biclustering and a GO tree search. Volcano plots of gene expression changes between MSI colon and endometrial cancers were constructed using the Enhanced Volcano Bioconductor package.^[25]^

### 2.3. Biclustering analysis and selection of cancer-specific biclusters

We conducted biclustering analysis of the normalized gene expression dataset using the MoSBi Bioconductor package.^[26]^ MoSBi provides an integrated interface for different frequently used biclustering algorithms, including FABIA,^[27]^ ISA,^[28]^ Plaid,^[29]^ and QUBIC,^[30]^ with default parameters for each algorithm. A bicluster is part of the entire dataset that consists of a subset of genes and samples with high correlation, which is defined based on the metrics used. Each algorithm produces different types of biclusters characterized by different features. To verify the similarity of the biclusters and the influence of the algorithm on this similarity, we assessed the Jaccard similarity between any 2 biclusters. In Jaccard similarity analysis, we defined the intersection between 2 biclusters A and B as a set of pairs consisting of gene and sample, where both genes and samples belong to both A and B, and the union as a set of pairs consisting of gene and sample, where both genes and samples belong to either A or B and then applied the definition of Jaccard similarity as follows:


$$\begin{array}{l} J\left(A,B\right)=\frac{\left|A⋂B\right|}{\left|A⋃B\right|} \end{array}$$

This value can be obtained in a straightforward manner for each pair of biclusters and is represented as a heatmap generated via hierarchical clustering. On the basis of visual inspection, the influence of the algorithms on Jaccard similarity can be readily identified. Subsequently, we did not perform any algorithm-specific analysis of the biclusters and combined these within a single dataset.

A cancer-specific bicluster was defined as a cluster in which sample distribution was significantly enriched for either MSI colon or endometrial cancer. To select cancer-specific biclusters, we used Fisher exact test (*q* value < 0.001 and odds ratio of either colon or endometrial cancer >1). These procedures accordingly facilitated the selection of biclusters closely associated with cancer. For example, if a bicluster consisted of only 55 colon cancer samples, it would have unique features present in a subset of MSI colon cancers, although not in the remainder of the samples related to the genes in the bicluster. However, the aforementioned example represents an extreme case, and given that our data were noisy, a suitable statistical measure is required. Fisher exact test is a simple statistical method applicable to this problem. The odds ratio shows the direction of data bias, and consequently, if in a comparison between a specific cancer (either colon or endometrium) and other conditions, the odds ratio is >1, it could be considered cancer (either colon or endometrium)-specific.

### 2.4. Functional analysis of DEGs and biclusters

To investigate the functional features of our DEGs and biclusters, and to select functionally relevant biclusters, we carried out GO enrichment analysis using gprolifer2.^[31]^ Among the enriched GO terms, we selected only those in the category of biological process (BP). In this regard, Gprolifer2, a widely used functional annotation tool and a programmatic interface using REST API, has previously been implemented, enabling researchers to construct automated analysis pipelines written in R.^[31]^ An adjusted *P* value of 0.05 was applied to select enriched GO terms for each DEG set and cancer-specific bicluster, and we presented the main results of GO enrichment graphically using AmiGO^[32]^ and REVIGO.^[33]^ The interactive graph created by REVIGO also depends on the European Bioinformatics Institute Gene Ontology Annotation database.^[34]^

### 2.5. Selection of functionally relevant biclusters using GO

To identify functionally relevant biclusters among cancer-specific biclusters using the GO enrichment results, we developed a novel method based on the relationships between the GO terms enriched with DEGs and those in the biclusters in the GO tree structure. We reasoned that if a bicluster was functionally more relevant to either MSI colon or endometrial cancers than to other conditions, then numerous enriched GO terms in the bicluster would be child terms of the enriched GO terms enriched with DEGs.

On the basis of these assumptions, we developed 2 metrics, namely, the term childness score and term-set childness score, the former of which provides a representation of the depth of GO a given term among the DEG GO terms and is formally defined as follows:

Term childness = the maximum distance of a query GO term to any parent GO term/(maximum distance of a query GO term to any parent GO term + maximum distance of a query GO term to any child GO term),

where parent and child terms indicate GO terms with either parent or child relationships in a type of relation, as either “is_a” or “part_of” relationships in the transition-enabled GO tree, and distance indicates the number of DEG GO terms involved in the transitive path from a query GO term to a DEG GO term. The transitive property indicates that if term A is a parent of term B, and term B is a parent of term C, then term A is a parent of term C. This transitive property also applies to the entire GO tree.

The term-set childness score is simply the mean score of the term childness score of terms belonging to a term set, which is generally the result of a GO enrichment analysis. On the basis of the distribution of the term-set childness score of the biclusters, we set the threshold of significance of the term-set childness score to 0.6. Although the threshold of 0.6 was more or less lenient when combined with the other filtering processes, it did not have any substantial influence on the final results obtained.

In these schemes, if a bicluster had a term-set childness score >0.6, it was deemed to be functionally relevant. On the basis of the term childness score thus obtained, a gene childness score was also derived as the maximal term childness score of all the term childness scores of all GO BP terms of an annotated gene.

By comparing the cancer and noncancer-specific biclusters, we assessed whether the childness score was a reasonable measure of functional relevance. Given that numerous child terms of DEGs were cancer-related and numerous normal genes were excluded from our DEG list, we predicted that childness scores would be higher in cancer-specific biclusters than in cancer-specific biclusters.

### 2.6. Survival analysis

To evaluate the functional significance of the selected functionally relevant biclusters as potential biomarkers, we performed survival analysis using TCGA clinical data for colon and endometrial cancers, which were directly linked to the samples in the biclusters. In survival analysis using functionally relevant biclusters, we assessed whether the significance of bicluster genes was limited to bicluster samples, and if not, whether this could be generalized to the entire dataset (either MSI colon or endometrial cancers). Specifically, for each functionally relevant bicluster, we obtained gene expression data consisting of the bicluster genes and total samples of either MSI colon or endometrial cancers, and then for those gene expression data, we performed PCA and stratified the data into 2 groups, and using 2-group stratification, we carried out survival analysis. The criteria used for the stratification using PCA were based on either the median value of the first or second principal component (PC1 or PC2) or K-means clustering (*K* = 2) of the PCA results. Survival analysis of the stratified data for each functionally relevant bicluster was performed using the Kaplan–Meier estimator for overall survival. Survival analysis entailed assessing whether functionally relevant biclusters were more significant than other biclusters.

## 3. Results

### 3.1. Identification of cancer-specific DEGs

**Figure 2.**
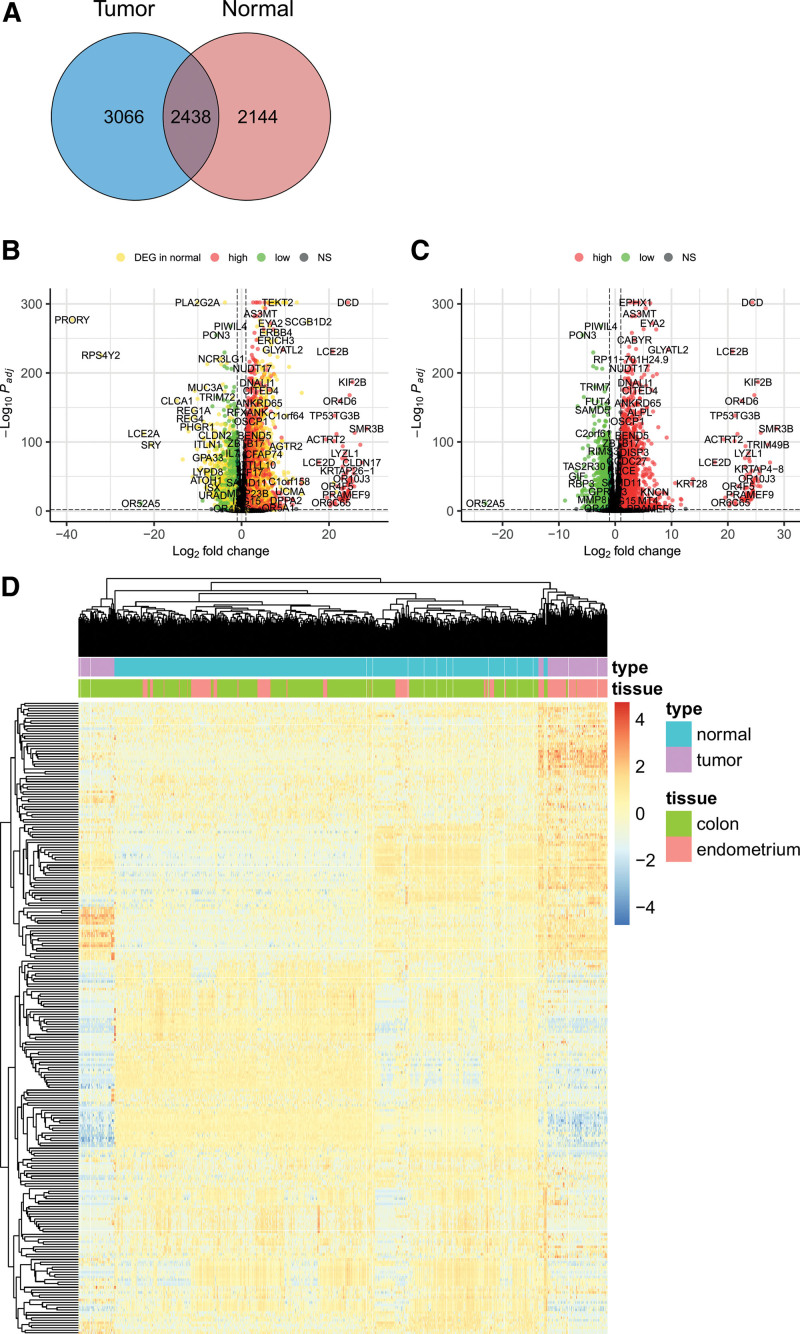
Selection of functionally relevant biclusters from cancer-specific biclusters using childness score. (A) The distribution of term childness score. The vertical dached line represents the threshold value (0.6). By definition, term childness score should be between 0 and 1. (B and C) Heatmap representation for illustration purposes of Bicluster 21, (B) functionally relevant biclusters, and that of (C) a subset of gene expression consisting of genes of Bicluster 21 and samples not belonging to the Bicluster 21. Expression values are not scaled to reveal the relationships between clustering tendency of genes (rows) and childness score of a gene. The childness score of a gene is adapted from the calculation of a term childness score. Among all the term childness scores from all the annotated GO biological process (BP) terms of a gene, the maximum value of the term childness score is defined as gene childness score. (D) Heatmap representation of Bicluster 1, an example of not functionally relevant biclusters. As in (B) and (C), the expression values are not scaled and the childness score of a gene is calculated in the same manner. GO = gene ontology.

Using DESeq2 with thresholds of an adjusted *P* value < 0.001 and absolute log2 fold change > 1, we identified 5504 protein-coding genes that showed differential expression between colon and endometrial MSI cancers. For normal samples, we identified 4582 DEGs using the same thresholds. Using these thresholds, we obtained 3066 cancer-specific DEGs from the 5504 tumor DEGs, excluding 2438 DEGs that were also identified in normal tissues (Fig. 2A). Among these, 2072 and 994 genes were up- and downregulated, respectively. A volcano plot of the tumor samples revealed that many of the genes differentially expressed in tumor samples were also differentially expressed in normal samples (Fig. 2B and C). Hierarchical clustering of the samples using cancer-specific DEGs revealed that tumor samples were well delineated between the colon and endometrium, whereas normal samples were heterogeneous, with the colon and endometrium mixed together (Fig. 2D).

### 3.2. Bicluster analysis

**Figure 3.**
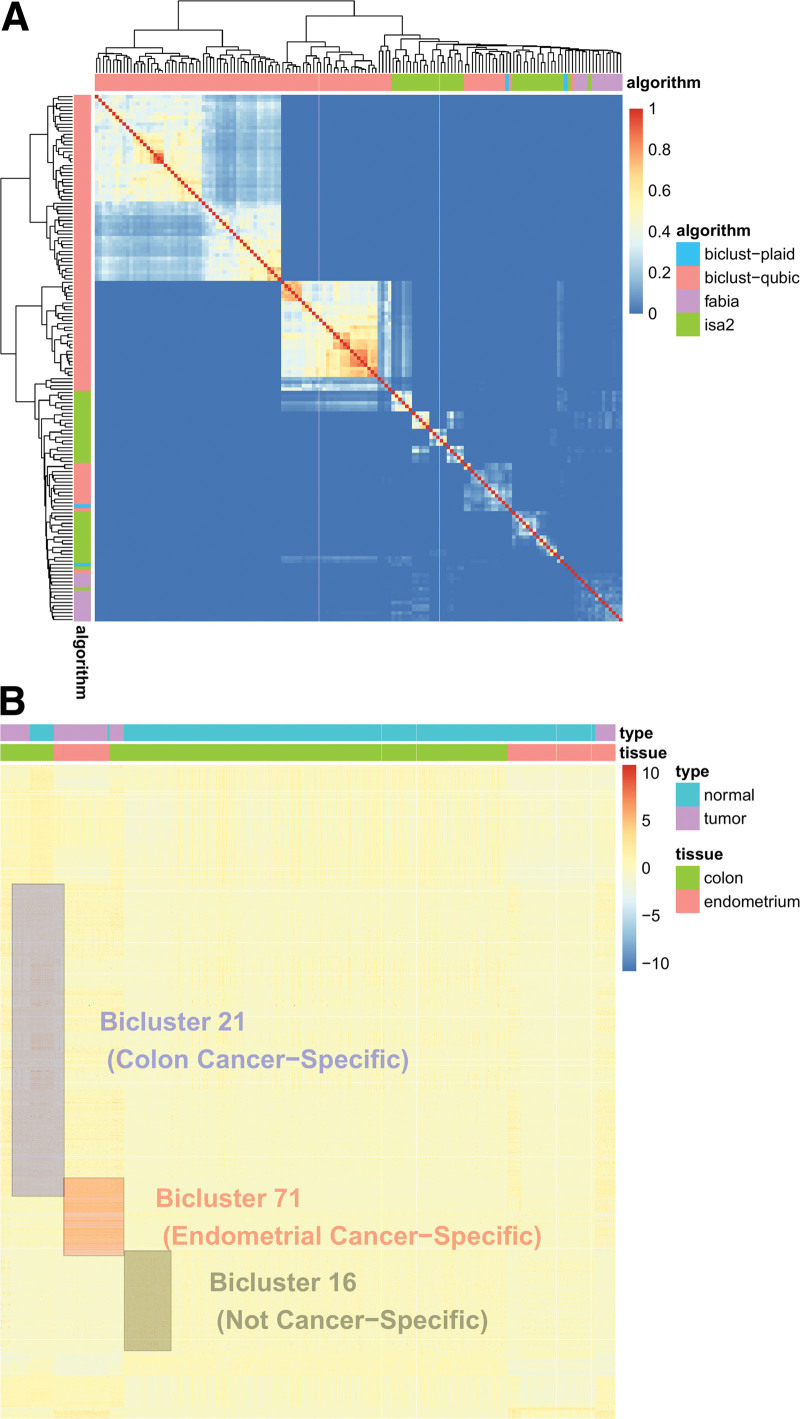
Graphical representation of differentially expressed genes (DEGs). (A) Venn diagram of DEGs of tumor (MSI colon vs endometrial cancers) and normal (colon vs endometrium) samples. DEGs were obtained using DESeq2 with adjusted *P* value <0.001 and absolute log2 fold change >1. (B and C) Volcano plot for the comparison between MSI colon and endometrial cancers. DEGs in normal samples were shown in (B) and removed in (C). The horizontal dashed line indicates adjusted *P* value = 0.001 and the vertical horizontal lines indicate log2 fold change = −1 and 1, respectively. The DEGs in the tumor samples with >1 log2 fold change are represented as red dots and those with <−1 log2 fold change as green dots. DEGs in the normal samples were represented as yellow dots regardless of fold change. (D) Heatmap of cancer-only DEGs with hierarchical clustering. Note that tumor samples are clearly delineated between colon and endometrial cancers while normal colon and endometrial tissues were relatively mixed together. DEGs = differentially expressed genes, MSI = microsatellite instability.

We used the MoSBi R/Bioconductor package to perform biclustering based on the 4 aforementioned algorithms, which yielded a total of 153 biclusters with variable numbers of genes and samples: 2 using Plaid, 100 using QUBIC, 13 using FABIA, and 38 using ISA2. Values for Jaccard similarity between any 2 biclusters were obtained and represented as a heatmap generated via hierarchical clustering (Fig. 3A). A majority of the biclusters thus obtained tended to cluster according to the algorithm, thereby implying that each algorithm captured specific features of gene expression. As it appeared that the application of no single algorithm produced more reasonable results than any of the others, we collected the results for all 4 algorithms in a single set.

Among the 153 biclusters, we selected 41 cancer-specific biclusters using Fisher exact test (*q* value < 0.001 and odds ratios of either colon or endometrial cancer >1), of which, 32 and 9 were enriched in colon cancer and endometrial cancers, respectively. Examples of the cancer-specific and noncancer-specific biclusters are shown in Figure 3B.

### 3.3. GO terms enriched with DEGs and cancer-specific clusters

**Figure 4.**
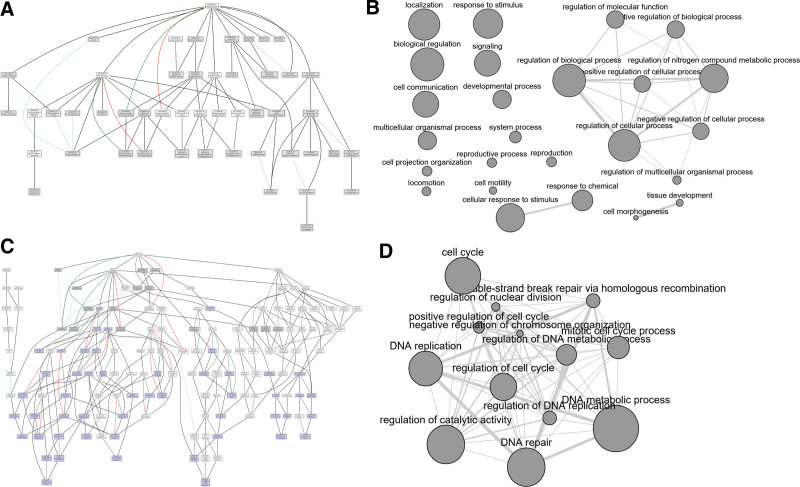
Graphical representation of biclustering results. (A) Heatmap representation with hierarchical clustering of Jaccard similarity between 153 biclusters. Refer to section 3 of Materials and Methods for the calculation of Jaccard similarity between a pair of biclusters. Note that the biclusters tend to cluster according to the algorithms. (B) Heatmap representation of 3 biclusters (Biclusters 21, 71, and 16) for illustration purposes. Sample composition of Biclusters 21, 71, and 15 is predominant by colon cancers, endometrial cancers, and normal colons, respectively. Note the small portion of overlap between Biclusters 21 and 71, and Biclusters 71 and 16. There are no overlaps between Biclusters 21 and 16 as represented in the heatmap.

We performed GO enrichment analysis for both DEGs and 41 cancer-specific biclusters using gprolifer2. Each significant GO term was investigated using QuickGO or AmiGO2.^[32,35]^ The top-ranked GO BP terms showing DEG enrichment included “multicellular organismal process,” “developmental process,” “anatomical structure development,” “regulation of cellular process,” “biological regulation,” and “cell differentiation,” many of which are general terms located around the root term of GO BP (Fig. 4A). However, these terms do not appear to provide useful information with respect to the cancer-specific differences between colon and endometrial MSI cancers. In contrast, a number of the cancer-specific biclusters tended to have cancer-related terms that were more specific to GO structure. For example, in bicluster 21, we detected enrichment of “cytokine-mediated signaling pathway,” “cell-cell junction organization,” “cellular response to cytokine stimulus,” “epithelial cell differentiation,” and “tyrosine phosphorylation of STAT protein,” and in bicluster 37, “regulation of kinase activity,” “DNA repair,” “regulation of DNA replication,” “regulation of kinase activity,” “regulation of chromosome organization,” and “cell cycle” were enriched (Fig. 4C). When the interactions among these terms were analyzed using REVIGO, we identified a more organized and consistent structure between bicluster 37-enriched terms than between DEG-enriched terms (Fig. 4B and D). Furthermore, we established that many of the enriched GO terms in bicluster 37 were child terms of the DEG-enriched terms. Our findings based on a manual inspection appeared to be generalizable and were further quantified and developed into childness scores.

### 3.4. Identification of functionally relevant biclusters

**Figure 5.**
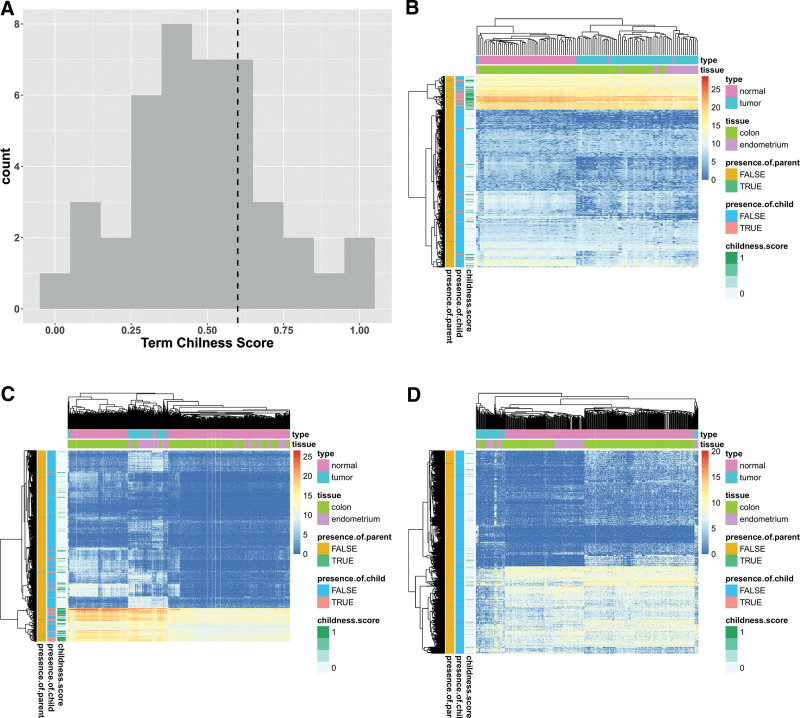
Graph representations of enriched GO BP terms in the DEGs and the bicluster genes. (A) 38 representative enriched GO terms in the DEGs. Grey rectangles represent DEG GO terms whereas white rectangles (not DEG GO terms) are shown to inform the paths from the DEG GO terms to the root GO term (“biological process”). The relationships between a pair of GO terms are represented as colored edges. Black indicates is_a relationship, blue part_of, red negatively_regulates, and green positively_regulates. (B) Interactive graph among representative enriched GO BP terms in the DEGs made by REVIGO. The size of a node represents the LogSize value (the frequency of the term in the EBI GOA database) of the term and the width of an edge represents the degree of semantic similarity of a pair of terms. (C) Thirty representative-enriched GO BP terms in genes in the Bicluster 37. Purple rectangles represent bicluster genes and gray rectangles represent DEG GO terms. White rectangles (neither bicluster nor DEG GO terms) are shown to inform the paths from either the bicluster GO terms or the DEG GO terms to the root GO term (“biological process”). The relationships between a pair of GO terms are represented as colored edges. Black indicates is_a relationship, blue part_of, red negatively_regulates, and green positively_regulates. (D) Interactive graph among representative enriched GO BP terms in the Bicluster 37 made by REVIGO. The size of a node represents the LogSize value (the frequency of the term in the EBI GOA database) of the term and the width of an edge represents the degree of semantic similarity of a pair of terms. BP = biological process, DEG = differentially expressed gene, EBI = European Bioinformatics Institute, GO = gene ontology, GOA = Gene Ontology Annotation.

Using novel childness scores, we sought to select functionally relevant biclusters from the cancer-specific biclusters The childness score reflects the extent to which GO terms in a bicluster tend to be child GO terms among DEGs. When we set the threshold of the childness score to 0.6 according to the distribution of childness scores (Fig. 5A), 8 biclusters were selected as functionally relevant biclusters from among the cancer-specific biclusters. When we compared the distribution of the term-set childness scores between the cancer-specific biclusters and the remainder of the biclusters using the Wilcoxon rank-sum test, we found that term-set childness scores in the cancer-specific biclusters were significantly higher than those in the noncancer-specific biclusters (*P* < .001). These findings tend to imply that many of the enriched GO terms in the cancer-specific biclusters are child terms of DEG GO terms, thereby indicating that the childness score might have applicability in the selection of functionally relevant biclusters.

When functionally relevant biclusters were further analyzed via hierarchical clustering, the samples (columns) of the biclusters tended to cluster according to the tissue type or whether they were cancerous or normal (Fig. 5B). Interestingly, some genes (rows) with high childness scores tended to cluster into a few gene clusters, and were typically characterized by a higher expression than other genes. Although these trends were not pronounced, we did detect them when the samples unassociated with the biclusters were examined, to say, to conduct the same hierarchical clustering for the matrix made by genes belonging to the biclusters but by the samples outside the biclusters (Fig. 5C). However, these trends were not identified in the nonfunctionally relevant biclusters (Fig. 5D).

### 3.5. Functionally relevant biclusters as potential biomarkers

**Figure 6.**
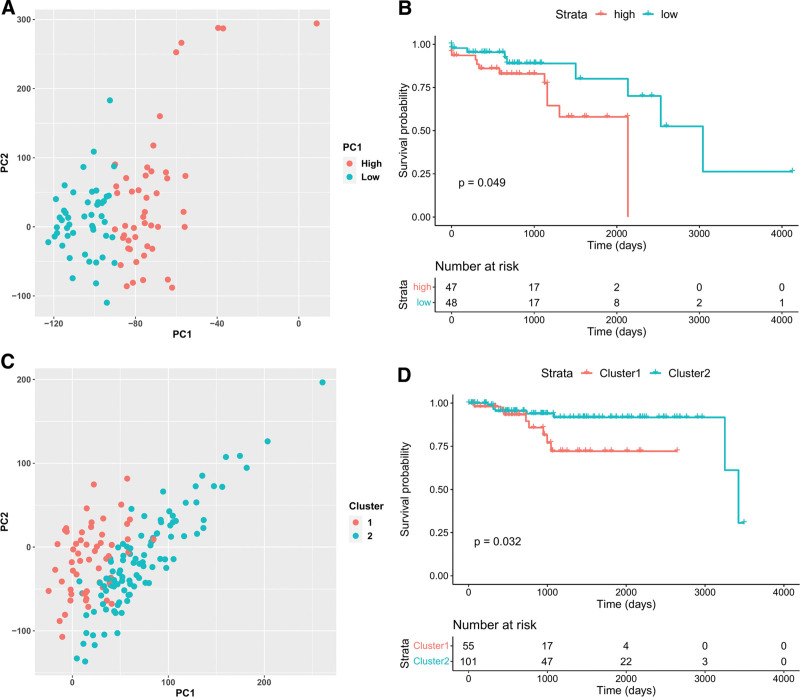
Survival analysis for the functionally relevant biclusters (Bicluster 12 & 89). (A) Principal component analysis (PCA) for the gene expression values of MSI colon cancer samples. PCA is performed only for the genes of Bicluster 12. MSI colon cancer samples are stratified into 2 groups according to PC1 values (high & low). (B) A survival curve (overall survival) of MSI colon cancer samples which are stratified into 2 groups (high & low) according to the PC1 values of PCA using genes of the Bicluster 21. (C) PCA of gene expression of MSI endometrial cancers. PCA is performed only for the genes of Bicluster 89. The samples are stratified into 2 groups according to the results of K-means clustering (Cluster 1 & 2). (D) A survival curve (overall survival) of MSI endometrial cancer samples which are stratified into 2 groups (cluster1 & cluster2) according to the K-means clustering results for the gene expression values of the genes of the Bicluster 89. MSI = microsatellite instability.

On the basis of survival analysis, we assessed the potential utility of functionally relevant biclusters as biomarkers. PCA of the gene expression of bicluster genes in the tumor samples, with tumor samples (either colon or endometrium) stratified into 2 groups according to the PCA results, revealed 6 (75%) of the 8 functionally relevant biclusters to be statistically significant for overall survival in either the colon or endometrial cancer cohort (Fig. 6A–D). In contrast, in the remaining biclusters, the likelihood of statistically significant identification in the survival analysis was considerably lower (28%). Statistically significant biclusters in survival analysis were rarely detected among random biclusters with the same number of genes and samples. Collectively, these findings indicate that functionally relevant biclusters may serve as clinically useful biomarkers.

## 4. Discussion

In this study, to identify candidate biomarkers for enhancing the therapeutic efficacy of MSI tumor treatment, we investigated genuine cancer-specific functional differences between 2 types of MSI cancers (colon and endometrial) based on error-prone DEG analysis. By combining DEG analysis and biclustering using the GO graph structure, among >100 biclusters, we identified 8 biclusters specific to the context, 6 of which showed clinical significance, as validated by survival analysis.

To the best of our knowledge, this is the first study in which the differential functional profiles of MSI colon and endometrial cancers have been assessed using gene expression data. To date, most transcriptomic studies on MSI colon and endometrial cancers have tended to focus on the differentiation of MSI from MSS cancers or on molecular subtyping,^[36–40]^ and we suspect that the lack of research addressing these issues can probably be attributed to the extreme methodological dependence of DEG analysis.

DEG analysis is the most frequently adopted approach used for gene expression profiling. However, the typically applied DEG analysis pipeline, which has been particularly effective in analyzing samples derived from the same tissue of origin, might be insufficient for identifying cancer-specific genes that are differentially expressed between cancers associated with different tissues or organs. Consequently, this may result in the prominence of false-positive genes representing tissue specificity between normal tissue types. However, to a certain extent, GO enrichment analysis appears to be resistant to the inclusion of false-positive genes, given that GO terms are enriched only when a sufficiently large number of genes are annotated to a particular term. On the basis of these properties, GO can be used to combine DEGs and biclusters, as in the present study. However, despite the robustness of GO enrichment analysis, further research should be conducted to develop methods for producing more accurate DEG results in samples with >1 type of interactions.

We anticipate that further investigation of the 8 functionally relevant biclusters identified in this study will provide insights into the distinct pathogenesis and therapeutic strategies for MSI colon and endometrial cancers (Table 1). Bicluster 53 is enriched with respect to the 2 GO terms “tube lumen cavitation and “salivary gland cavitation, which are child GO terms of the DEG term, “anatomical structure formation.”^[41]^ MSI colon tumors tend to be poorly differentiated, implying that tube lumen formation is relatively weak, whereas MSI endometrial cancer tends to be an endometrial type characterized by preserved tube formation, although poorly differentiated types predominate.^[42,43]^ These findings thus indicate that tube-forming properties, which may be mediated by the EDAR or SHH protein,^[44,45]^ are important functional differences between MSI colon and endometrial cancers, of which, EDAR has been established to promote colorectal carcinogenesis via the Wnt/β-catenin signaling pathway.^[46]^ It is notable that in the DEG list, of the 2 genes SHH and EDAR in bicluster 53 annotated to the term “tube lumen cavitation,” only EDAR was included, which would be insufficient to enrich this GO term. Consequently, these important features of MSI tumors would not have been identified based solely on DEG analysis.

**Table 1:** Summary of functionally relevant biclusters.

Bicluster ID	Number of genes	Enriched GO terms	Representative GO terms in biclusters	Representative genes in biclusters	Significance in survival analysis (overall survival)
Bicluster 9	839	10	GO:0007186 (G protein-coupled receptor signaling pathway)	*GALP, MCHR2, NTS, NTSR1, NXPH3, NXPH4, PCSK1N, RELA, TAC3, UTS2R, BDKRB2, FZD4, FZD10, GPR176, OR2AG2, OR5F1,OR6A2,PDE3A,VAV1,XPR1*	Not significant
			GO:0007218 (neuropeptide signaling pathway)		
Bicluster 12	6196	83	GO:0007188 (adenylate cyclase-modulating G protein-coupled receptor signaling pathway)	*ADCY4,GCGR,GLP2R,GNA14,GNA15,MC4R,PTH2R,SCTR,VIPR1,WASF2, BPIFA1,CAMP,CXCL8,DEFB127,FAU,GALP,H2BC10,HMGN2,REG1A,RNASE7*	Significant
			GO:0061844 (antimicrobial humoral immune response mediated by antimicrobial peptide)		
Bicluster 21	838	24	GO:0007260 (tyrosine phosphorylation of STAT protein)	*CAV1,CNTF,FER,HPX,IFNL4,IL2,IL15,IL21,JAK1,JAK2,JAK3, BMP2,BMPR1A,BMPR1B,BMPR2,FRS2,GTF2F1,MAP2K6,MAPK11,MAPK14,RUNX2*	Significant
			GO:0019221 (cytokine-mediated signaling pathway)		
Bicluster 45	20,702	1590	GO:0002263 (cell activation involved in immune response)	*AR,CYP7B1,EGFR,FLCN,HLX,HSF4,KDR,KIT,NKX2-5,PSEN1, CLEC4E,CX3CR1,FOXP1,IL6,MLH1,PYCARD,RASGRP1,SBNO2,SLC11A1,STAT3*	Not significant
			GO:0050673 (epithelial cell proliferation)		
Bicluster 46	20,600	1543	GO:0030307 (positive regulation of cell growth)	*BCL2,DNPH1,H3-3A,KDM2B,LGI1,MTOR,PSMD10,RICTOR,SFRP2,ZNF639, AREG,EDN1,EMX1,EXT1,KDM5B,SEMA3A,SPG11,TBX2,TSC22D4,WNT7B*	Significant
			GO:0060560 (developmental growth involved in morphogenesis)		
Bicluster 47	20,695	1550	GO:0002042 (cell migration involved in sprouting angiogenesis)	*ADTRP,ITGB1,KDR,MIA3,NR4A1,NRP1,ROBO1,SRF,TDGF1,VEGFA, ADAM10,CD46,GALNT11,LLGL2,POFUT1,RFNG,SREBF2,SYNJ2BP,TSPAN15,YJEFN3*	Significant
			GO:0008593 (regulation of Notch signaling pathway)		
Bicluster 53	175	2	GO:0060605 (tube lumen cavitation)	*EDA,EDAR,NFIB,SHH,TGM2*	Significant
			GO:0060662 (salivary gland cavitation)		
Bicluster 89	88	2	GO:0035092 (sperm DNA condensation)	*HI-7,H2BC1,KAT5,PRM1,PSME4,RNF8,SYCP1,SYCP3,TNP2,TSSK6*	Significant
			GO:0035093 (spermatogenesis, exchange of chromosomal proteins)		

GO = gene ontology.

Frequently identified cancer-related GO terms including “transforming growth factor beta receptor signaling pathway” (biclusters 46 and 47), “G protein-coupled receptor signaling pathway” (bicluster 12), or “tyrosine phosphorylation of STAT protein” (bicluster 21) were also identified in some functionally relevant biclusters. Among these cancer-related pathways, the TGF-β pathway has been found to be more frequently mutated in MSI colorectal cancer than in MSI endometrial cancer.^[47]^ It is interesting that SMAD4, a member of the TGF- β pathway, was reported to be associated with a poorer prognosis in MSI-H CRCs, but not in MSS CRCs.^[48,49]^ In endometrial cancers, however, the differential roles of SMAD4 in the prognosis depending on MSI status were not reported.

The contribution of the JAK-STAT and G protein-related pathways to the carcinogenesis of colon and endometrial cancers is substantial, although little is currently known regarding their differential functions in colon and endometrial cancers.^[50,51]^ Although, to the best of our knowledge, the MSI-specific features of these pathways have yet to be reported, our novel findings provide evidence to indicate that there might be differences between MSI colon and endometrial cancers with respect to the degree of the contribution of these pathways.

Subtle differences in immune-related functions, which are believed to exist between the 2 highly immunogenic tumors, were also observed in some biclusters.^[52]^ Immune-related GO terms included “antimicrobial humoral response” (bicluster 12), “cytokine-mediated signaling pathway” (bicluster 21), and “T cell activation” (biclusters 46 and 47), and it is conceivable that the fine regulation of these immune-related functions might contribute to the maximal therapeutic efficacy of immune checkpoint inhibitors in MSI tumors.

Some enriched GO terms were found to be difficult to interpret despite the rigorous effort. For example, bicluster 89 was enriched with respect to “sperm DNA condensation and “spermatogenesis and exchange of chromosomal proteins, which are not believed to have a direct association with cancer. Despite the lack of interpretability, certain cancer-related genes, such as CDH5, were identified, which are annotated to “spermatogenesis.”^[53]^ However, further studies are required to determine the “missing link” between spermatogenesis and carcinogenesis.

Certain DEGs were notable in that they are believed to be biologically relevant. Among these, DCD is a secreted protein that promotes cell growth and survival, particularly under conditions of oxidative stress, and serves as an important MSI marker in CRC.^[54]^ However, little is currently known regarding MSI at the DCD site or its gene expression. In the present study, we found that compared with CRC, DCD showed a large fold change (log2 fold change: 11.7) in endometrial carcinoma (Fig. 3C), and an increase in DCD gene expression has been established to be associated with poor survival in breast cancer.^[55]^ Considering the generally better prognosis of MSI-CRC than that of endometrial carcinoma, these findings are interesting. Further studies are necessary to determine whether the invasiveness of DCD is also applicable to MSI-CRC and endometrial carcinomas.

PON3 is a member of the paraoxonase (PON) gene family characterized by antioxidant properties.^[56]^ It has been established that in endometrial cancers, MSI events are more frequent in the 3′ untranslated region of PON3, than in CRC.^[57]^ As in the case of DCD, little is known regarding the association between the MSI events and gene expression of PON3. In the present study, we obtained a log2 fold change value of −5.8 for the comparison between endometrial cancer and CRC, thereby indicating a considerably higher expression in CRC (Fig. 3C). In hepatocellular carcinoma, PON3 has been demonstrated to inhibit cancer cell proliferation, and a reduction in PON3 expression is associated with shorter disease-free and overall survival.^[58]^ However, the role of PON3 in MSI cancers needs to be further elucidated.

Although gene expression profiling is an essential step in functional profiling, caution should be exercised to avoid overemphasizing the importance of gene expression profiling, particularly when the tissues of origin differ, as in the present study. The main functional differences between MSI colon and endometrial cancers could be attributable to multiple factors, including not only tissue properties, which may be reflected by gene expression, but also gross anatomy, histological composition, and physiology, thereby making it difficult to precisely characterize gene expression. Given that this study was primarily based on gene expression profiling alone, further studies will be necessary to investigate more holistic systems, including gene expression, anatomy, and physiology.

The limitations of our research are closely associated with those of current bicluster search algorithms. As shown in Figure 4A, the biclustering results of the individual biclustering algorithms are highly clustered, thereby tending to imply the occurrence of pronounced algorithm-specific bias. We believe that this bias would contribute to preventing the discovery of a larger number of functionally relevant biclusters than the 8 identified in this study. With improvements in biclustering search methods, we would anticipate that a more extensive range of functionally relevant biclusters of diagnostic and therapeutic significance will be discovered.

Fine-tuning data preprocessing prior to running biclustering algorithms could also contribute to enhancing bicluster selection. Specifically, in this study, we did not consider the predominance of normal samples in our dataset, and the data were entered as input data for biclustering without correcting for the predominance of normal samples. The bias in the sample-type distribution toward normal samples may thus have contributed to a preferential selection of normally predominant biclusters. Further studies are accordingly necessary to minimize the impact of sample-type bias on biclustering. Nevertheless, we believe that our crude method is effective, at least to a certain extent. Moreover, the strong cancer-specific features of our functionally relevant biclusters enabled us to overcome sample bias and refine the biclusters, which consisted primarily of cancer samples.

Our research also provides a foundation for the integration of multi-omics data using biclusters. By extending our research using the multi-omics data, we would identify the regulators of biclusters at the mutation, copy number variation, or microRNA levels. If the interactions between the regulators and gene expression are proven to be biologically relevant, these findings will validate our observations in the present study. In this regard, we plan to conduct a multi-omics study on biclusters as a separate research project.

In conclusion, in this study, we examined the functional differences between MSI colon and endometrial cancers based on gene expression profiling, combining DEG analysis and biclustering in a context-relevant manner. More functionally relevant biclusters that can be used as diagnostic and therapeutic biomarkers were obtained via an indirect combination of DEG results and biclustering. We also addressed the issues relating to measures that could be adopted to enhance our novel workflow. This workflow is not limited to the functional profiling of MSI colon and endometrial cancers, and in conjunction with an expansion of the active applications, we anticipate that a more extensive range of clinically applicable biomarkers will be identified.

## Author contributions

**Conceptualization:** Woong Na, Young Soo Song, Sung Hak Lee.

**Formal analysis:** Woong Na, Il Ju Lee, Young Soo Song.

**Funding acquisition:** Young Soo Song.

**Investigation:** Woong Na, Sung Hak Lee.

**Methodology:** Woong Na, Il Ju Lee, Young Soo Song.

**Supervision:** Insong Ko, Mihye Kwon, Sung Hak Lee.

**Validation:** Woong Na, Il Ju Lee.

**Visualization:** Woong Na, Il Ju Lee, Young Soo Song.

**Writing – original draft:** Woong Na, Il Ju Lee, Young Soo Song, Sung Hak Lee.

**Writing – review & editing:** Woong Na, Insong Ko, Mihye Kwon, Young Soo Song, Sung Hak Lee.

## Supplementary Material


