# Supplementary material for: Cancer-specific functional profiling in microsatellite-unstable (MSI) colon and endometrial cancers using combined differentially expressed genes and biclustering analysis
Source: Medicine (Baltimore). 2023 May 12;102(19):e33647. doi: 10.1097/MD.0000000000033647 (PMC10174364; doi:10.1097/MD.0000000000033647)

Figure 1. Pincipal component analysis (PCA) of gene expression of TCGA normal and GTEx samples. TCGA samples include normal colon (COAD), rectum (READ), and endometrium (UCEC). GTEx samples include transverse colon (Colon–Transverse), sigmoid colon (Colon-Sigmoid) and uterus (Uterus). X axis represents principal component 1 (PC1) and Y axis principal component 2 (PC2). Each point represents a PCA-transformed sample which is color-coded according to the tissue origin. Each color-coded ellipse represent the region that contains 95% of samples of the group under the hypothetical Gaussian distribution.

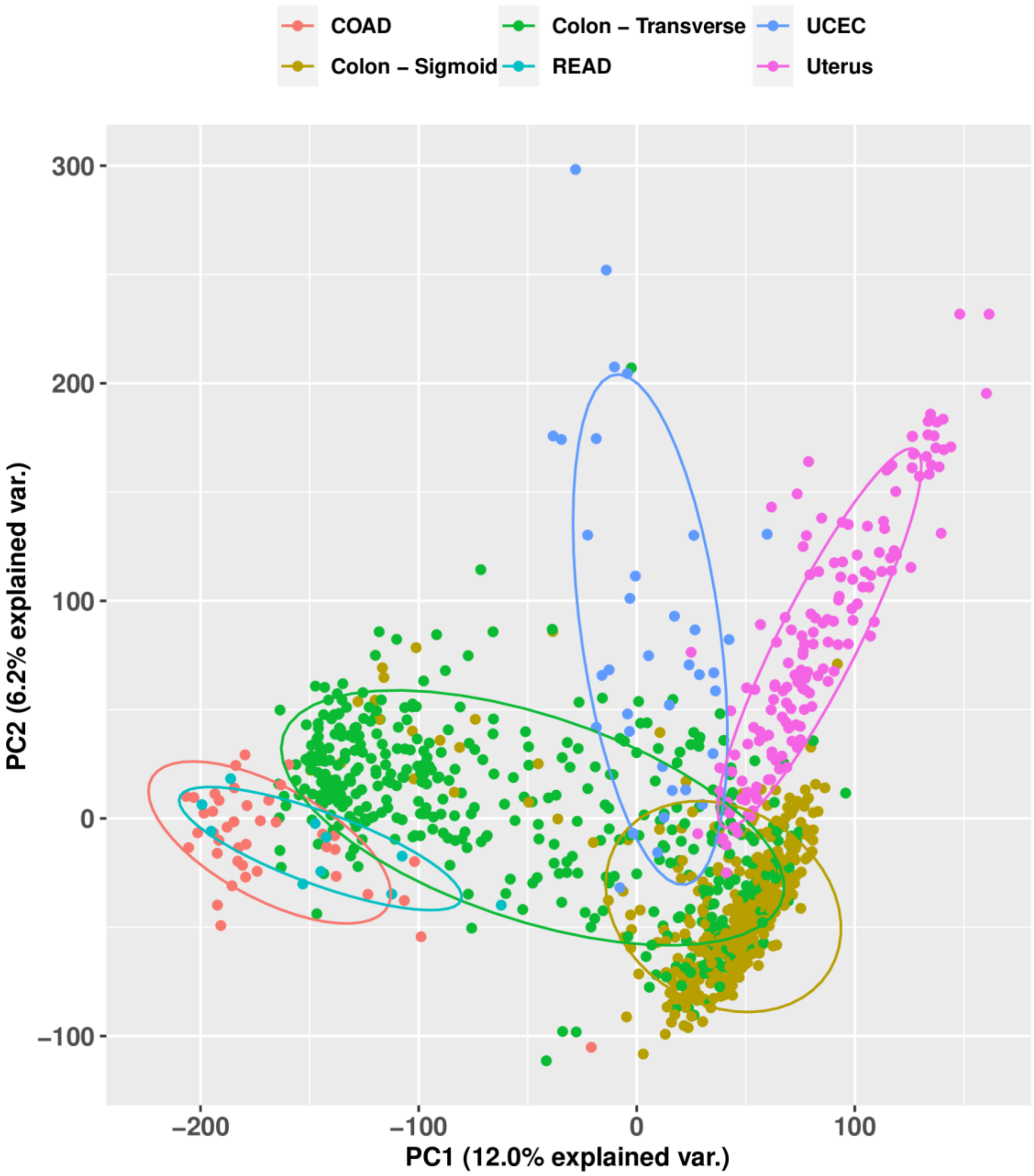

Supplement: Supplementary file 1 [file medi-102-e33647-s001.pdf]
